# Argon Inhalation Attenuates Retinal Apoptosis after Ischemia/Reperfusion Injury in a Time- and Dose-Dependent Manner in Rats

**DOI:** 10.1371/journal.pone.0115984

**Published:** 2014-12-23

**Authors:** Felix Ulbrich, Nils Schallner, Mark Coburn, Torsten Loop, Wolf Alexander Lagrèze, Julia Biermann, Ulrich Goebel

**Affiliations:** 1 Department of Anesthesiology and Intensive Care Medicine, University Medical Center, Freiburg, Germany; 2 Department of Anesthesiology, University Hospital RWTH Aachen, Aachen, Germany; 3 Eye Center, University Medical Center, Freiburg, Germany; Virginia Commonwealth University, United States of America

## Abstract

**Purpose:**

Retinal ischemia and reperfusion injuries (IRI) permanently affect neuronal tissue and function by apoptosis and inflammation due to the limited regenerative potential of neurons. Recently, evidence emerged that the noble gas Argon exerts protective properties, while lacking any detrimental or adverse effects. We hypothesized that Argon inhalation after IRI would exert antiapoptotic effects in the retina, thereby protecting retinal ganglion cells (RGC) of the rat's eye.

**Methods:**

IRI was performed on the left eyes of rats (n = 8) with or without inhaled Argon postconditioning (25, 50 and 75 Vol%) for 1 hour immediately or delayed after ischemia (i.e. 1.5 and 3 hours). Retinal tissue was harvested after 24 hours to analyze mRNA and protein expression of Bcl-2, Bax and Caspase-3, NF-κB. Densities of fluorogold-prelabeled RGCs were analyzed 7 days after injury in whole-mounts. Histological tissue samples were prepared for immunohistochemistry and blood was analyzed regarding systemic effects of Argon or IRI. Statistics were performed using One-Way ANOVA.

**Results:**

IRI induced RGC loss was reduced by Argon 75 Vol% inhalation and was dose-dependently attenuated by lower concentrations, or by delayed Argon inhalation (1504±300 vs. 2761±257; p<0.001). Moreover, Argon inhibited Bax and Bcl-2 mRNA expression significantly (Bax: 1.64±0.30 vs. 0.78±0.29 and Bcl-2: 2.07±0.29 vs. 0.99±0.22; both p<0.01), as well as caspase-3 cleavage (1.91±0.46 vs. 1.05±0.36; p<0.001). Expression of NF-κB was attenuated significantly. Immunohistochemistry revealed an affection of Müller cells and astrocytes. In addition, IRI induced leukocytosis was reduced significantly after Argon inhalation at 75 Vol%.

**Conclusion:**

Immediate and delayed Argon postconditioning protects IRI induced apoptotic loss of RGC in a time- and dose-dependent manner, possibly mediated by the inhibition of NF-κB. Further studies need to evaluate Argon's possible role as a therapeutic option.

## Introduction

Ischemia and reperfusion injuries (IRI) play a crucial role in the pathophysiology of various diseases such as diabetic retinopathy [1], retinal vascular occlusion [2] or anterior optic neuropathy [3], and possibly glaucoma [4], [5]. IRI ultimately leads to neuronal death via apoptosis [6] or necrosis [7]. Among the various neuronal cells, which are characterized in the retina, retinal ganglion cells (RGCs) are the ones most vulnerable to ischemia [8]–[10]. Clinical evaluation of many neuroprotective strategies only showed inconsistent tissue protection or none at all [11].

Argon - one of the noble gases - just emerged into scientific focus due to its organoprotective properties. Although discovered in 1885 by Sir William Ramsay and Lord Rayleigh, the first description of Argon's biological activity dated in 1930 and it was not until 1998, when Russian researches found first evidence for a possible tolerance of mice against hypoxic environmental conditions under Argon exposition [12]. Since then, Argon has been subjected to a few models of organ dysfunction or injury, aiming towards protection at different levels. Argon has recently been shown to protect organ function including kidney- and heart protection [13]–[16] and neuronal cells through anti-inflammatory effects possibly including GABA and NMDA receptors in a model of experimental brain injury [17]–[20]. While Argon is very easy to apply, there are no anesthetic or unwanted effects [21].

However, the neuroprotective effects and the responsible mechanisms of Argon on neuronal tissue *in vivo*, in particular on retinal ganglion cells (RGC), have not been investigated. The RGC represent a special population of neuronal tissue, as they are positioned “upstream” of the central nervous system, easily accessible, and treatable under direct visual control. Retinal IRI has been used as a brain injury model to prove various neuroprotective interventions before [22]–[24]. Although only 90% of the RGC receive the dye via the superior colliculi, these cells represent a fairly good amount of neuronal cells responsible for visual effects in the rats eye. The remaining 10% of RGC (among which are melanopsin ganglion cells) are not labeled with the fluorogold dye and are thus not counted in this experimental setting. Because of the neurons limited potential in regeneration, their protection and the therapy of neuronal injuries in cells and organ systems is one main area of research. Therefore, we chose RGC – respectively the inner retina – to analyze and counteract IRI related neuronal injury [10], [25], [26]. Postconditioning is related to a way, where subcritical ischemia [27] or volatile anesthetics [28] can exert cytoprotection in neuronal cells even after IRI and thus represents a strategy with high clinical relevance.

We hypothesized that Argon postconditioning exerts neuroprotective effects in IRI by reduced apoptosis in a time- and dose-dependent manner.

## Materials and Methods

### Animals

Adult male and female Sprague-Dawley rats (1∶1, 280–350 g bodyweight, Charles River, Sulzfeld, Germany) were used in these experiments. Animals were fed with a standard diet *ad libitum*, being kept on a 12-h light/12-h dark cycle. All procedures involving the animals concurred with the statement of The Association for Research in Vision and Ophthalmology for the use of animals in research and were approved a priori by the Committee of Animal Care of the University of Freiburg (Permit No: 35-9185.81/G-12/110). All types of surgery and manipulations were performed as previously described [10], [25], [26]. The number of animals used for RGC quantification and molecular analysis was n = 8 per group. For analysis of mRNA- and protein-expression retinal tissue was harvested at t = 24 h after Argon inhalation.

### Retrograde labeling of RGC

Sprague Dawley rats were anesthetized with isoflurane and placed in a stereotactic apparatus (Stoelting, Kiel, Germany). The skin overlying the skull was cut open und retracted. The lambda and bregma sutures served as landmarks for drilling 3 holes on each site of the bregma sutures. A total amount of 7.8 µl fluorogold (FG) (Fluorochrome, Denver, CO, USA) dissolved in DMSO/PBS was injected into both superior colliculi through the drilling holes [29]. 7 days prior to further experimental intervention were allowed for adequate retrograde transport of FG into the RGC's perikarya.

#### Retinal ischemia/reperfusion injury and Argon treatment

Following randomization, rats were sedated intraperitoneally and the anterior chamber of the left eye was cannulated with a 30-gauge needle connected to a reservoir containing 0.9% NaCl. Intraocular pressure was increased to 120 mm Hg for 60 minutes and ocular ischemia was confirmed microscopically by interruption of the retinal circulation. Reperfusion was initiated by removing the needle tip promptly. Rats without immediate recovery of retinal perfusion at the end of the ischemic period or those with lens injuries were excluded from the investigation, since the latter prevents RGC death and promotes axonal regeneration [30]. To evaluate a neuroprotective effect of inhalative Argon, animals were randomized to receive treatment either with room air or with Argon 25, 50 or 75 Vol% in 21% oxygen and the respective amount of nitrogen (54% for Argon 25 Vol%, 29% for Argon 50 Vol% and 4% for Argon 75 Vol%; Air Liquide, Kornwestheim, Germany) for 60 minutes in an air-sealed chamber (dimension of chamber: 20*35*27 cm; flow rate 8 L/min initially for minute for 10 minutes – then reduced to 2 L/min). Concentration of Argon were composed and tested by the supplier. Postconditioning treatment was either initiated immediately following retinal IRI or with a delay of 1.5 or 3 hours.

#### RGC quantification

Animals were sacrificed by CO_2_-inhalation 7 days after ischemia. Retinal tissue was immediately harvested, placed in ice-cold Hank's balanced salt solution and further processed for whole mount preparation. Retinae were carefully placed on a nitrocellulose membrane with the ganglion cell layer (GCL) on top. After removing the vitreous body, retinae were fixed in 4% paraformaldehyde for 1 h and then embedded in mounting media (Vectashield; Axxora, Loerrach, Germany). The densities of FG-positive RGC were determined in blinded fashion using a fluorescence microscope (AxioImager; Carl Zeiss, Jena, Germany) and the appropriate bandpass emission filter (FG: excitation/emission, 331/418 nm), as previously described [10], [25], [26]. Briefly, we photographed 3 standard rectangular areas (0.200 mm×0.200 mm = 0.04 mm^2^) at 1, 2 and 3 mm from the optic disc in the central regions of each retinal quadrant. Hence, we evaluated an area of 0.48 mm^2^ per retina. To calculate the average RGC density in cells/mm^2^, we multiplied the number of analyzed cells/0.04 mm^2^ with 25. Secondary fluorogold stained activated microglia cells (AMC) after RGC phagocytosis were identified by morphologic criteria and excluded from calculation. All data are presented as mean RGC densities [cells/mm^2^] ± SD.

### Immunohistochemical staining

The eyes (n = 3) were enucleated 7 days after ischemia, embedded in Tissue-Tek (Sakura-Finetek, Torrance, CA), and frozen in liquid nitrogen. Frozen sections (10 µm) were cut through the middle one-third of the eye and collected on gelatinized slides. Immunohistochemistry was performed according to standardized protocols [10]. The following monoclonal antibody was used: mouse anti-GFAP (glial fibrillary acidic protein; Neomarkers #AB-1GA5), which is upregulated in astrocytes and Müller cells under various environmental stress conditions [31]. The antibody was then conjugated with the corresponding secondary antibody (Alexa Fluor 568, Life Technologies). The nuclei of retinal cells were stained by adding 4′,6-diamino-2-phenylindole dihydrochloride hydrate (DAPI; Sigma) to the Mowiol embedding medium. Slides were examined under a fluorescence microscope (Axiophot, Carl Zeiss).

### Western blot analysis

24 h after ischemia retinal tissue for analysis of protein expression was harvested. Total protein from ¾ of retina was extracted and processed for Western Blot as described previously [32]. The membranes were blocked with 5% skim milk in Tween20/PBS and incubated in the recommended dilution of protein specific antibody (Cleaved Caspase-3 #9664 and p-NF-κB #8242, Cell Signaling Technology, Danvers, MA, USA) overnight at 4°C. After incubation with a horseradish peroxidase-conjugated anti-rabbit secondary antibody (GE Healthcare, Freiburg, Germany), proteins were visualized using the ECL plus Chemiluminescence Kit (GE Healthcare). For normalization, blots were re-probed with Caspase-3 (#9665) and ß-Actin (#4967S) or total NF-κB (#3033, all Cell Signaling Technology, Danvers, MA, USA). Relative changes in protein expression in I/R injured retinas either with or without Argon were calculated in relation to the corresponding non-ischemic retinas.

### Real time polymerase chain reaction (RT-PCR)

From retinal tissue harvested 24 h after ischemia, total RNA from ¼ of retina was extracted using a column-purification based kit (RNeasy Micro Kit, Qiagen, Hilden, Germany) according to the manufacturer's instructions. Reverse transcription was performed with 50 ng of total RNA using random primers (High Capacity cDNA Reverse Transcription Kit, Applied Biosystems, Darmstadt, Germany). Real time polymerase chain reactions (RT-PCR) were done with TaqMan probe-based detection kit (TaqMan PCR universal mastermix, Applied Biosystems, Darmstadt, Germany). Following primers were used: Bax #Rn02532082_g1, Bcl-2 #Rn99999125_m1, Caspase-3 #Rn00563902_m1, NF-κB #Rn01399565_m1 (all from Applied Biosystems, Darmstadt, Germany). The PCR assays were then performed on a RT-PCR System (ABI Prism 7000, Applied Biosystems, Darmstadt, Germany) with the following cycling conditions: 95°C for 10 min, 40 cycles of 95°C for 10 sec and 60°C for 1 min. Reaction specificity was confirmed by running appropriate negative controls. Cycle threshold (CT) values for each gene of interest were normalized to the corresponding CT values for GAPDH (ΔCT). Relative gene expression in I/R injured retinal tissue either with Argon or room air was calculated in relation to the corresponding gene expression in the non-injured retinal tissue of each individual animal (ΔΔCT).

### Whole blood analysis

Whole blood samples of the animals were analyzed using the Siemens Advia 120 Hematology System and a white blood cell count (WBC) was performed. Data are expressed as cell counts (×10^3^/µL).

### Statistical analysis

Data was analyzed using a computerized statistical program (SigmaPlot Version 11.0, Systat Software Inc., San Jose, CA, USA). We wished to detect a 50% reduction of IRI through CO intervention. Based on previously published data [10], [25], [26] we assumed that a sample size of eight animals per group would be sufficient to detect such reduction. The results are presented as means (±SD) after normal distribution of data had been verified. One-way ANOVA for repeated measurements was used for between-group comparisons with post hoc Tukey Kramer test and Kruskal Wallis test for data with lack of normal distribution. *P*<0.05 was considered statistically significant.

## Results

### Argon inhalation time- and dose-dependently protects RGC from IRI induced apoptosis

**Figure 1:**
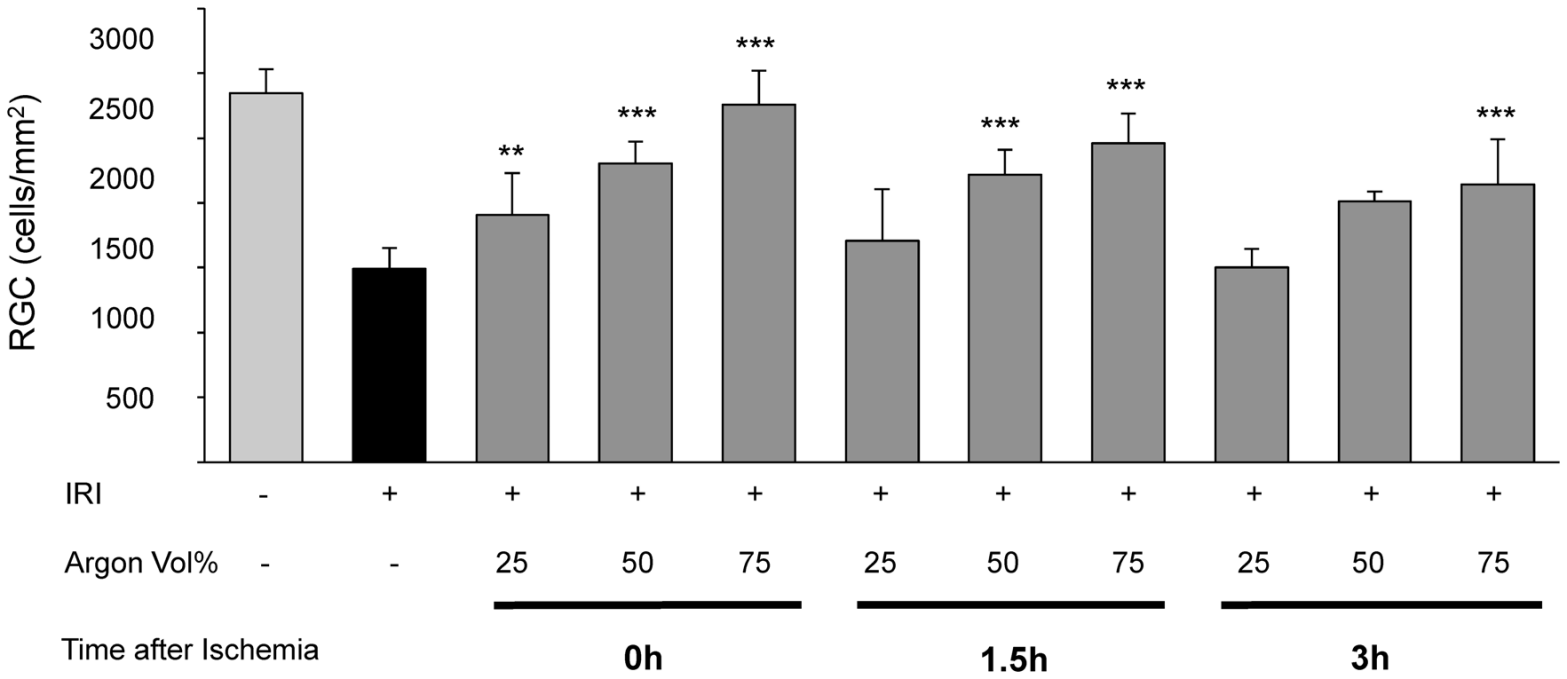
Time- and dose-dependent influence of Argon inhalation on retinal ganglion cell count after ischemia reperfusion injury (IRI). Quantification of retinal ganglion cell density (cells/mm^2^, data are mean±SD, n = 8, ***  = p<0.001 IRI vs. IRI+Argon 75 Vol% at all time points, vs. IRI+Argon 50 Vol% at 1.5 h and 3 h delayed treatment, **  = p<0.01 IRI vs. IRI+Argon 25 Vol% immediate treatment).

Retinal IRI damage led to a density loss of approximately 50% RGCs (IRI 1504±300 vs. untreated 2846±400; Fig. 1, Col. 1 and 2). Argon inhalation after IRI diminished this effect time- and dose-dependent (Fig. 1, Col. 3–11).

**Figure 2:**
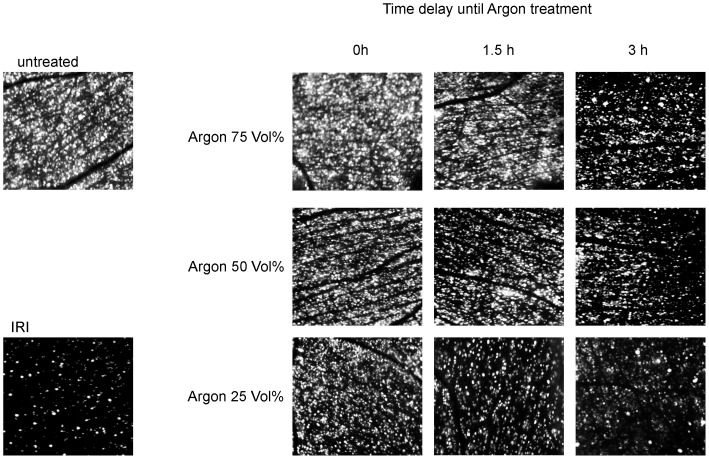
Time- and dose-dependent influence of Argon inhalation on vital retinal ganglion cells after ischemia reperfusion injury (IRI). Representative flat mount images (n = 8) of fluorogold labeled retinal ganglion cells 7 days after IRI and immediate and delayed (i.e. 1,5 and 3 hours delay) Argon inhalation (25, 50 and 75 Vol%).

Inhalation of Argon 25Vol% immediately after IRI had a slight, but significant protective effect for RCG loss (IRI+Argon 25Vol% immediately: 1908±325; p<0.01). Inhalation of Argon 50Vol% immediately after IRI showed a significant lower retinal damage compared to IRI, although to a lesser extent than Argon 75 Vol% at the same timepoint (IRI+Argon 50Vol% immediately: 2268±325 and IRI+Argon 75Vol% immediately 2761±257; both p<0.001). Inhalation of Argon 25Vol% administered with a delay of 1.5 h after IRI did not have a protective effect. In contrast, inhalation of Argon 50Vol% 1.5 h after IRI showed a significant lower retinal damage compared to IRI, although to a lesser extent than Argon 75 Vol% at the same timepoint (IRI+Argon 50Vol% 1.5 h after IRI: 2151±275 and IRI+Argon 75Vol% 1.5 h after IRI: 2463±225; both p<0.001). Inhalation of Argon 25 and 50Vol% with a delay of 3 hours after ischemia had no effect regarding RGC loss. Delayed exposure of Argon 75Vol% (3 h) after IRI mediated a significant reduction of retinal cell loss (IRI+Argon 75Vol% 3 h after IRI: 2143±350; p<0.001). Representative pictures of the IRI induced loss of vital RGCs and Argon's effects are shown in Fig. 2.

### Argon inhalation reduces IRI induces retinal caspase-3 mRNA expression and caspase-3 cleavage

**Figure 3:**
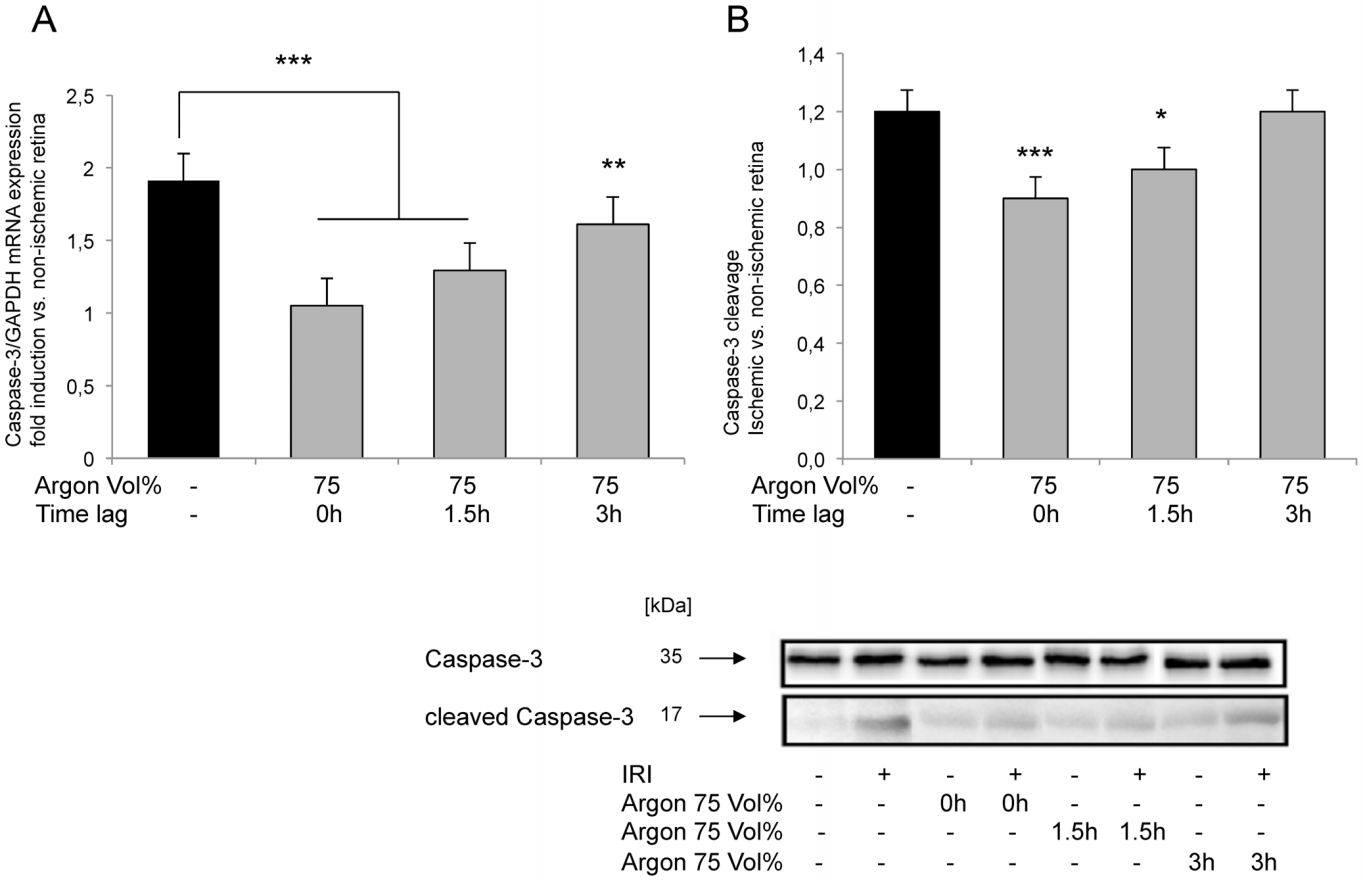
Effect of Argon inhalation on retinal expression of Caspase-3 mRNA and Caspase-3 cleavage. (**A**) Fold induction of Caspase-3 mRNA expression after time-dependent inhalation of Argon 75 Vol% in ischemic retinal tissue compared to GAPDH in relation to the corresponding non-ischemic retinae analyzed by RT-PCR (n = 8; data are mean±SD; ***  = p<0.001 IRI vs. IRI+Argon immediately and at 1.5 h delayed treatment and **  = p<0.01 IRI vs. IRI+Argon at 3 h delayed treatment). (**B**) Densitometric analysis of n = 8 western blots for caspase-3 cleavage after time-dependent inhalation of Argon 75 Vol% (data are mean±SD; ***  = p<0.001 IRI vs. IRI+Argon immediately and *  = p<0.05 IRI vs. IRI+Argon at 3 h delayed treatment) and representative western blot image (n = 8) showing the suppression of retinal cleavage of caspase-3 compared to uncleaved caspase-3.

Inhalation of Argon 25 and 50Vol% after retinal IRI did not show significant changes regarding caspase-3 mRNA expression or Caspase-3 cleavage at any time (data not shown). Immediate inhalation of Argon 75Vol% reduced both Caspase-3 mRNA expression and cleavage of Caspase-3 compared to untreated control animals (Fig. 3A: IRI 1.91±0.46 vs. IRI+Argon 75Vol% immediately 1.05±0.36 and Fig. 3B: IRI 1.23±0.11 vs. IRI+Argon 75 Vol% immediate 0.95±0.13, both p<0.001). Fig. 3 shows the fold-induction of Caspase-3 mRNA expression after time-dependent inhalation of Argon 75 Vol% in ischemic retinal tissue compared to GAPDH in relation to the corresponding non-ischemic retinae analyzed by RT-PCR (n = 8; data are mean±SD; ***  = p<0.001 IRI vs. IRI+Argon immediately and at 1.5 h delayed treatment and **  = p<0.01 IRI vs. IRI+Argon at 3 h delayed treatment). Moreover, a densitometric analysis of n = 8 western blots for caspase-3 cleavage after time-dependent inhalation of Argon 75 Vol% (data are mean±SD; ***  = p<0.001 IRI vs. IRI+Argon immediately and *  = p<0.05 IRI vs. IRI+Argon at 3 h delayed treatment) and representative western blot image (n = 8) showing the suppression of retinal cleavage of caspase-3 compared to uncleaved caspase-3. Significant changes were also detectable after a delayed inhalation of 1.5 h (Fig. 3A: IRI+Argon 75Vol% 1.29±0.47; p<0.001 and Fig. 3B: IRI+Argon 75Vol% 0.95±0.13, p<0.05). Inhalation 3 h after IRI resulted in a significant difference in mRNA-expression, while caspase-3 cleavage was not altered (Fig. 3A: IRI+Argon 75Vol% 3 h after IRI 1.61±0.38, p<0.01).

### Argon inhalation inhibits IRI induced Bcl-2 and Bax expression

**Figure 4:**
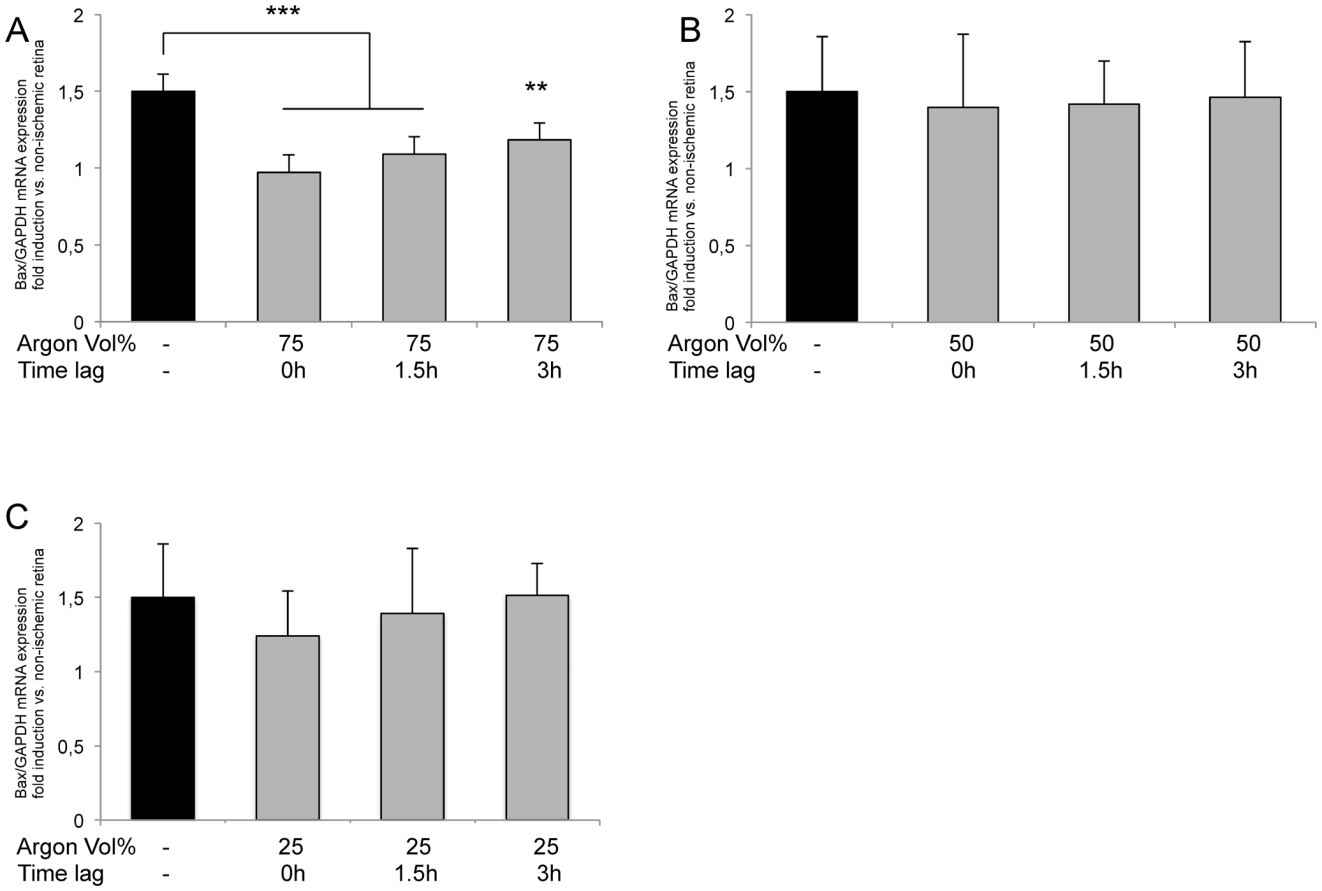
Effect of Argon inhalation on retinal expression of Bax mRNA. Fold induction of Bax mRNA expression in ischemic retinal tissue compared to GAPDH in relation to the corresponding non-ischemic retinae analyzed by RT-PCR after (A) 75 Vol% Argon inhalation, (B) 50 Vol% Argon inhalation and (C) 25 Vol% Argon inhalation; (n = 8; data are mean±SD; ***  = p<0.001 IRI vs. IRI+Argon 75 Vol% immediately and IRI+Argon 75 Vol% at 1.5 h delayed treatment, **  = p<0.01 IRI vs. IRI+Argon 75 Vol% at 3 h delayed treatment).

Expression of Bax was dose-dependently reduced by Argon inhalation depending on the application dose (Fig. 4A–C). Immediate inhalation of Argon 75Vol% after IRI showed the most intense effect (IRI 1.64±0.30 vs. IRI+Argon 75Vol% immediately 0.78±0.29; p<0.001). Delayed administration of Argon (1.5 and 3 h) still significantly decreased Bax expression, but to a lesser degree (IRI+Argon 75Vol% at 1.5 h after IRI: 0.89±0.22; p<0.001 and Argon 75Vol% at 3 h after IRI: 1.09±0.19; p<0.01). Inhalation of Argon 25 or 50Vol% was not associated with a significant reduction of Bax expression.

**Figure 5:**
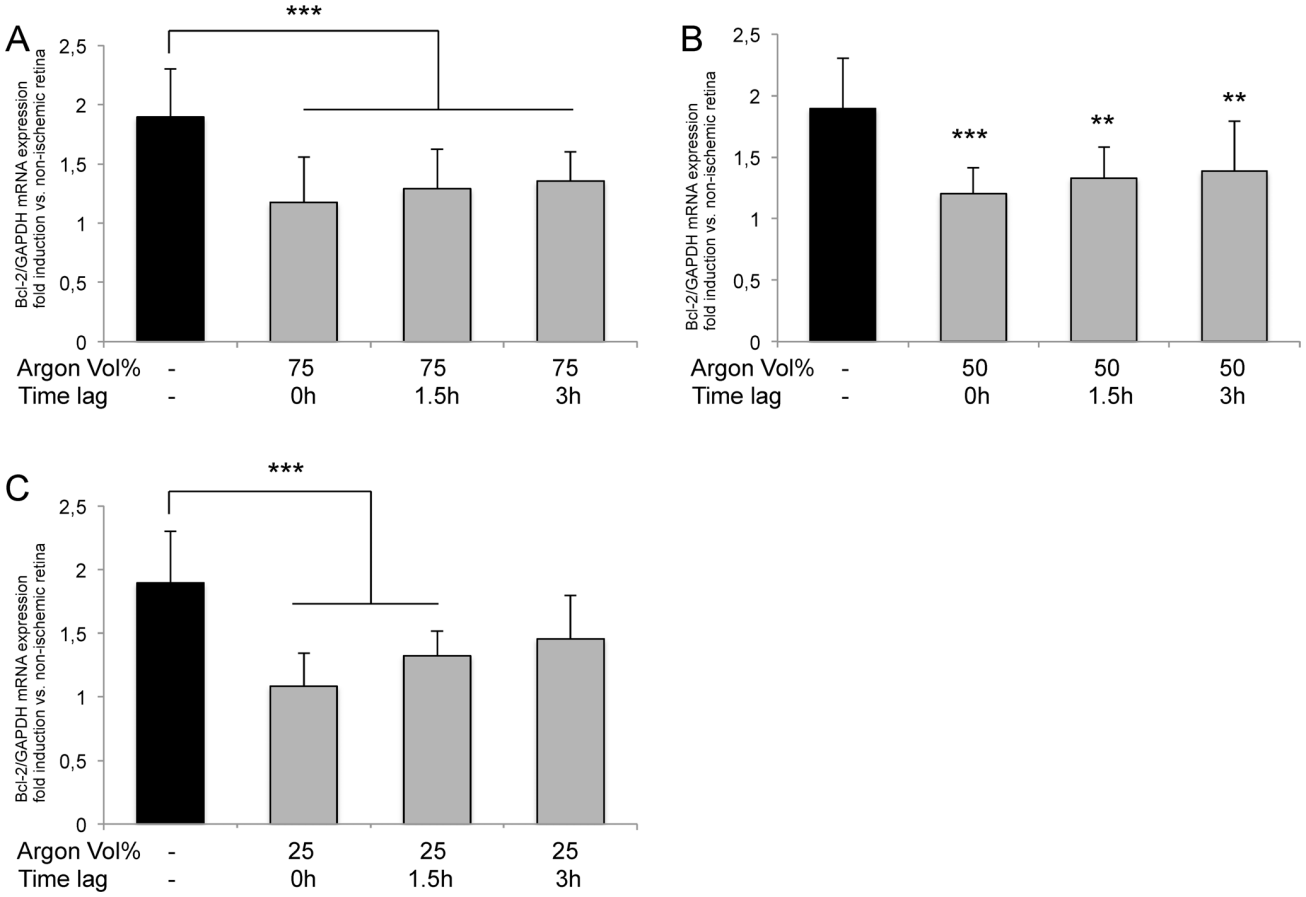
Effect of Argon inhalation on retinal expression of Bcl-2 mRNA. Fold induction of Bcl-2 mRNA expression in ischemic retinal tissue compared to GAPDH in relation to the corresponding non-ischemic retinae analyzed by RT-PCR after (**A**) 75 Vol% Argon inhalation (***  = p<0.001 IRI vs. IRI+Argon at all time points), (**B**) 50 Vol% Argon inhalation (*** = p<0.001 IRI vs. IRI+Argon immediate treatment, **  = p<0.01 IRI vs. IRI+Argon at 1.5 h and 3 h delayed treatment) and (**C**) 25 Vol% Argon inhalation (***  = p<0.001 IRI vs. IRI+Argon immediately and at 1.5 h delayed treatment). All n = 8; data are mean±SD.

To our surprise, the expression of Bcl-2 was significantly reduced by Argon in all three concentrations and at almost every time point (Fig. 5A–C) compared to room air treated animals. Inhalation of Argon 75Vol% after IRI exhibited the most intense diminution of all time points (IRI 2.07±0.29 vs. IRI+Argon 75Vol% immediately: 0.99±0.22, IRI+Argon 75Vol% 1.5 h after IRI: 1.20±0.38 and IRI+Argon 75Vol% 3 h after IRI: 1.28±0.23; all p<0.001). Significant effects on Bcl-2 expression were also detectable with Argon 50Vol% and 25Vol%, if administered immediately or with a delay of 1.5 h after IRI.

### Argon inhalation attenuates white blood cell count in peripheral blood

**Figure 6:**
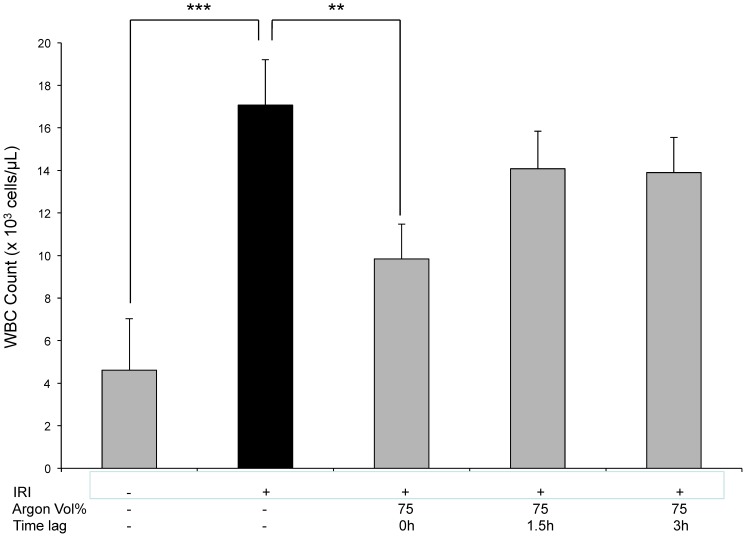
White blood cell count in peripheral blood after IRI and Argon postconditioning. (n = 8; data are expressed as mean cell counts (×10^3^/µL)±SD; ***  = p<0.001 untreated vs. IRI and **  = p<0.01 IRI vs. IRI+Argon immediately).

To answer the question whether retinal IRI has any influence concerning peripheral WBC count in terms of systemic inflammation in response to retinal ischemia and the role of Argon in this setting, we performed a whole blood analysis. While IRI resulted in a significantly elevated number of white blood cells (untreated animals 4,6±2,4*10^3^ cells/µL vs. IRI 17,1±2,1; p<0.001), immediate Argon inhalation (75 Vol%) lead to a significant decrease (Fig. 6: IRI 17,1±2,1 vs. IRI+Argon 75 Vol% immediately 9,8±1,6; p<0.01). No significant effects of Argon at any other time point or lower concentrations were detectable.

### Argon inhalation affects Müller cell activation

**Figure 7:**
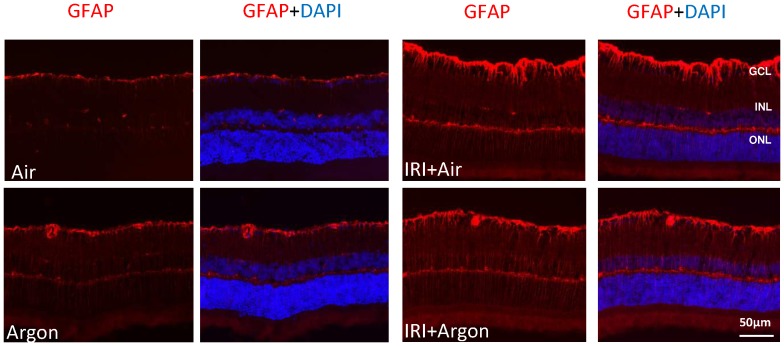
GFAP expression in the retina after unilateral IRI and Argon inhalation. Cross-sections of the retinae 7 days after unilateral IRI. In controls with and without immediate Argon 75 Vol% inhalation, GFAP was only positive in Müller cells and astrocytes in the GCL. After ischemia, GFAP was upregulated in Müller cells. Their processes, extending through all retinal layers, became GFAP positive. Scale bar, 50 µm.

In nonischemic animals with and without Argon postconditioning, GFAP was only positive in Müller cells' endfeet and astrocytes in the GCL (Fig. 7). After IRI, GFAP staining seems brighter in the GCL and to a somewhat lesser extent after Argon inhalation. In both groups, the Müller cells' processes, extending through all retinal layers, became GFAP positive. Following IRI, a band of positive staining is present in the OPL, which is not present in control retinae.

### Argon postconditioning suppresses NF-κB mRNA expression and phosphorylation

**Figure 8:**
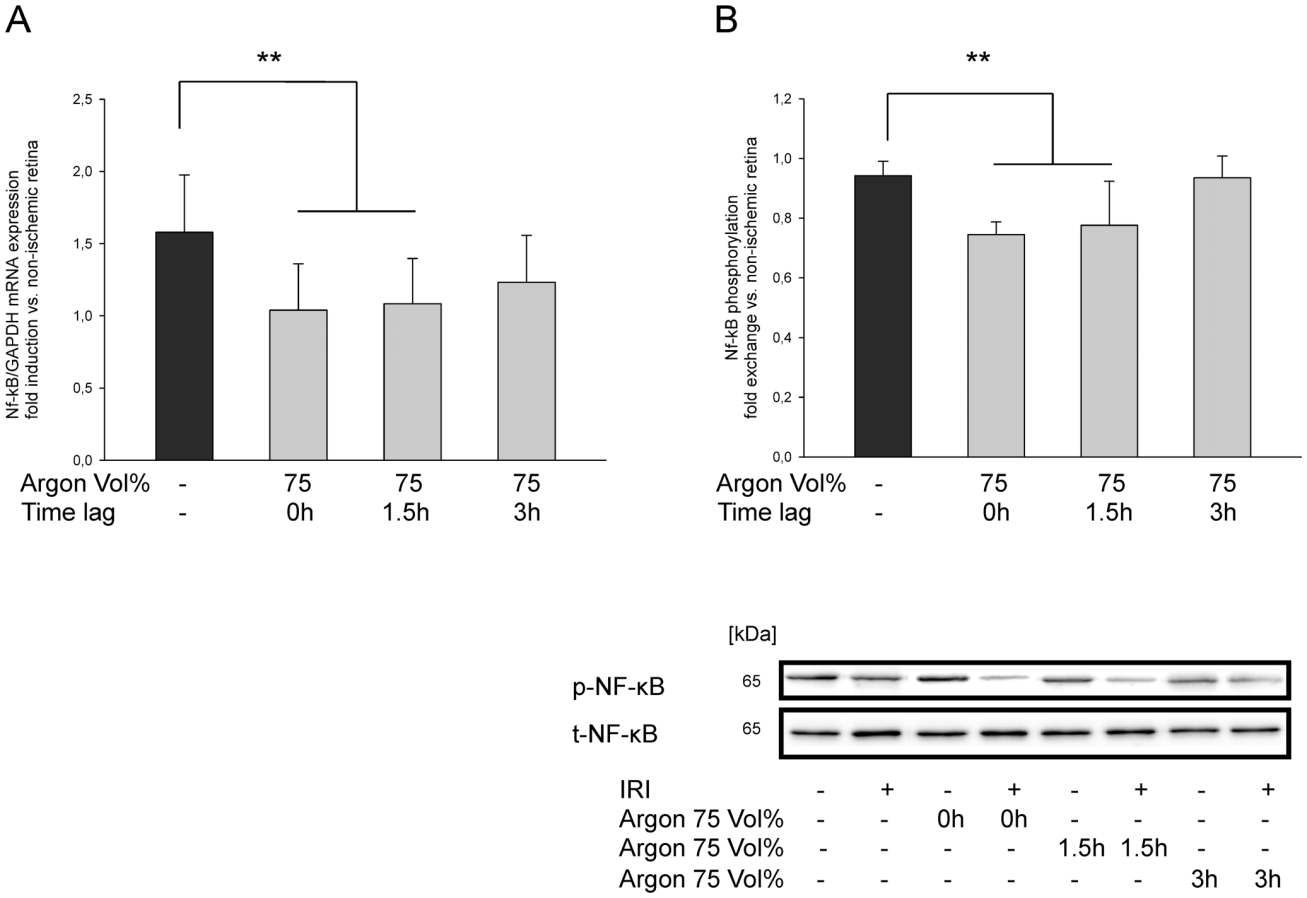
Effect of Argon inhalation on retinal expression of NF-κB mRNA and NF-κB phosphorylation. (**A**) Fold induction of NF-κB mRNA expression after time-dependent inhalation of Argon 75 Vol% in ischemic retinal tissue compared to GAPDH in relation to the corresponding non-ischemic retinae analyzed by RT-PCR (n = 8; data are mean±SD; **  = p<0.01 IRI vs. IRI+Argon at all time points). (**B**) Densitometric analysis of n = 8 western blots for NF-κB phosphorylation after time-dependent inhalation of Argon 75 Vol% (data are mean±SD; ***  = p<0.01 IRI vs. IRI+Argon immediately and at 1.5 h delayed treatment) and representative western blot image (n = 8) showing the suppression of retinal NF-κB phosphorylation compared to total NF-κB expression.

In order to analyze the pathway by which Argon mediates its effects, we assessed the influence of Argon inhalation regarding retinal NF-κB phosphorylation and expression. Argon 75Vol% attenuates retinal NF-κB mRNA expression and protein phosphorylation in a time-dependent manner. Immediate inhalation and 1.5 h delay after IRI reduced mRNA (Fig. 8A: IRI 1.58±0.40 vs. IRI+Argon 75Vol% immediately: 1.04±0.32 and IRI+Argon 75Vol% at 1.5 h after IRI: 1.08±0.31; both p<0.01) Accordingly, NF-κB phosphorylation was suppressed (Fig. 8B: IRI 0.94±0.05 vs. IRI+Argon 75Vol% immediately: 0.74±0.04 and IRI+Argon 75Vol% at 1.5 h after IRI: 0.78±0.15; both p<0.01). After 3 h delay no significant changes were detectable. Inhalation of Argon 25 and 50Vol% after retinal IRI did not show significant changes regarding NF-κB mRNA expression or phosphorylation at any time (data not shown).

## Discussion

We investigated the neuroprotective effects of Argon inhalation at different concentration and time points after retinal ischemia-reperfusion injury in rats. The major findings of this *in-vivo* study can be summarized as follows: (1) ischemia and reperfusion injury resulted in a significant loss of RGC, as shown by the decreased number of vital RGCs and induction of apoptosis. (2) The inhalation of Argon after IRI protected RGC in a linear time- and dose-dependent manner. (3) Argon postconditioning inhibited Bax and Bcl-2 mRNA expression as well as caspase-3 mRNA expression and cleavage. (4) A possible molecular mechanism of Argon-mediated protection may be the suppression of the transcription factor NF-κB. (5) Interestingly, we found, that Argon postconditioning attenuated the IRI-mediated rise of white blood cells in peripheral blood. These findings support our hypothesis that postconditioning with Argon exerts neuroprotection by suppressing apoptosis and thus providing cytoprotective effects after injury in neuronal organs.

While preconditioning may be used to protect organs in which ischemia is anticipated, postconditioning describes the phenomenon by which the function of organs - that have been subjected to sublethal ischemic periods - may be restored using various stimuli such as volatile agents [27], [33]. As previously reported, postconditioning with carbon monoxide (or corresponding releasing molecules) attenuates retinal IRI [10], [25], [34]. In this study, IRI resulted in a loss of vital RGC around 48%. Inhalation of Argon 75 Vol% immediately after IRI completely reduced the ischemic effect. Dose-reduction of Argon inhalation resulted in a decreased protective effect; nonetheless, even Argon 25 Vol% administered immediately after IRI still showed significant protective properties. Delayed Argon inhalation (1.5 or 3 hours) after IRI still resulted in significant protection. These results are in accordance with the studies of Loetscher et al. and Ryang et al., who showed a protection of neurons after cerebral ischemia and traumatic brain injury *in-vitro* (with immediate Argon treatment) and a reduction of infarct size after mid-artery occlusion *in-vivo* [20], [35]. Zhuang and coworkers reported a reduction of 42% in infarction size in a model of neonatal asphyxia in rats, when subjected to 70% Argon 2 hours after asphyxia [36]. In contrast to Ryang, who analyzed 24 hours after insult only, our results demonstrate a lasting neuroprotection for 7 days after ischemia [20].

Of note, Argon 75 Vol% did not only show the strongest protective effect, but also the most lasting ones, regarding delayed onset of therapy. Even if inhaled 3 hours after IRI, sustained neuroprotective effects are still significant, while Argon 50 Vol% is only protective if inhaled 1.5 hours after IRI and Argon 25 Vol% is only protective, if inhaled immediately. It is tempting to speculate, that Argon exerts its most potent and lasting effects at a high concentration only, while lower concentration may not start up cellular processes at a degree that exerts prolonged neuroprotection. So far no data for Argon is available to support the theory of dose- and time-dependency. In contrast, the noble gas Xenon has been proven to show a time-dependent effect regarding neuroprotection in a stroke model [37].

Previous studies in various *in-vivo* or *in-vitro* injury models have shown that Argon is able to reduce neuronal cell loss by a decrease in LDH release or an increase in total number of vital cells [38], [39]. Zhuang et al reported an Argon (70 Vol%) mediated increase in Bax, Bcl-2 and Bcl-xL protein expression in neonatal rats [36]. In contrast, our results provide evidence, that Argon reduces Bax and Bcl-2 mRNA expression time- and dose-dependently. While Bax is known to promote apoptotic cell death, the role of Bcl-2 may depend on its localisation (cytosolic or bound to mitochrondrial membrane) and its state of heterodimerisation [40]. As the cytosolic forms of all members of the Bcl family (including Bax) are inactive [41], [42], we choose the mRNA expression of these proteins to show metabolic rate and usage in the context of IRI and Argon postconditioning. As a consequence of this downregulation, effector caspase-3 mRNA expression and cleavage is significantly reduced – thus providing proof for a reduction in neuronal apoptosis. Although significantly, the lower levels of Bcl-2 and Bax may merely reflect less cell damage, as Argon is thought to act upstream of these two mediators.

The transcription factor NF-κB is a key regulator in inflammation and apoptosis in all cells and has been proposed as a possible target to treat neuronal ischemia [43]. Inhibition of NF-κB may result in a downregulation of Bcl-2-family proteins [44], [45]. In the present work, the mRNA expression was supressed and the phosphorylation of the p65-NF-κB subunit was attenuated by Argon (75 Vol%) in a time-dependent manner up to three hours. Interestingly, a certain (unknown) threshold of constitutive NF-κB activation is needed; otherwise neuronal survival may be impaired [46], [47]. The Argon mediated inhibition of NF-κB may at least be a possible molecular mechanisms of the downregulation of apoptotic proteins.

Immunohistochemistry results confirmed an affection of Müller cells and astrocytes after IRI. If this phenomenon is in accordance with the lesser degree of neuronal cell death needs to be analysed in future experiments, as well as the question if IRI or Argon directly influence retinal glia cell activation.

Interestingly, we found a reduced leukocyte count in peripheral blood 24 hours after IRI due to immediate Argon postconditioning (75 Vol%). This result was not hypothesis driven and thus must be judged very carefully in the context of argon's dose-dependent and mainly anti-apoptotic and not anti-inflammatory effects. According to clinical studies, an increased WBC is associated with poorer outcome after ischemia and reperfusion injury of the brain [48]. This is in accordance with experimental data regarding secondary brain injury [49].

Most recently, David and coworkers demonstrated that Argon increases the thrombolytic efficiency of tissue-plasminogen activator [50]. One might speculate that the reperfusion injury is lower due to better rheological properties, thus providing a more sufficient re-oxygenation to the retinal neurons.

In summary, our results show that Argon inhalation is a protective intervention to be used for neuronal postconditioning in a dose- and time-dependent manner. Argon inhalation at high doses and fast onset after IRI may reduce IRI damage by antagonizing pro-apoptotic induction. Moreover, Argon showed significant reduction of the peripheral white blood cell count, which may be a hint for the reduction of systemic inflammation. These effects may be mediated by inhibition of the transcription factor NF-κB. Further studies are needed to analyze the exact mechanisms of Argon's action.
